# A Mobile App for Postoperative Pain Management Among Older Veterans Undergoing Total Knee Arthroplasty: Mixed Methods Feasibility and Acceptability Pilot Study

**DOI:** 10.2196/50116

**Published:** 2023-10-18

**Authors:** Jessica Kelley Morgan, Caitlin R Rawlins, Steven K Walther, Andrew Harvey, Annmarie O'Donnell, Marla Greene, Troy G Schmidt

**Affiliations:** 1 Continuous Precision Medicine Research Triangle Park, NC United States; 2 Western North Carolina Veterans Affairs Health Care System Asheville, NC United States

**Keywords:** mobile app, pain management, opioids, older adults, veterans, mobile phone

## Abstract

**Background:**

Prescription opioid misuse risk is disproportionate among veterans; military veterans wounded in combat misuse prescription opioids at an even higher rate (46.2%). Opioid misuse is costly in terms of morbidity, mortality, and humanitarian and economic burden and costs the Civilian Health and Medical Program of the Department of Veterans Affairs more than US $1.13 billion annually. Preventing opioid misuse at the time of prescription is a critical component in the response to the opioid crisis. The CPMRx mobile app has been shown to decrease the odds of opioid misuse during the postoperative period.

**Objective:**

The overarching purpose of this feasibility pilot study was to explore whether deploying a mobile app (CPMRx) to track postoperative pain and medication use is feasible in a Department of Veterans Affairs medical center. In support of this goal, we had four complementary specific aims: (1) determine the technological and logistical feasibility of the mobile app, (2) assess the acceptability of the mobile app to participants, (3) measure demand for and engagement with the mobile app, and (4) explore the potential use of the mobile app to patients and providers.

**Methods:**

Participants (N=10) were veterans undergoing total knee arthroplasty within the Veterans Health Administration provided with the CPMRx app to self-manage their pain during their 7-day at-home recovery following surgery. CPMRx uses scientifically validated tools to help clinicians understand how a patient can use the least amount of medication while getting the most benefit. The suite of software includes a mobile app for patients that includes a behavioral health intervention and a clinical decision support tool for health care providers that provides feedback about pain and medication use trends. Patients filled out paper questionnaires regarding acceptability at their postoperative follow-up appointment.

**Results:**

Overall, quantitative measures of acceptability were high. The average rating for the amount of time required to use the app was 4.9 of 5 (5=“very little”), and the average rating for ease of use was 4.4 of 5 (5=“very easy”). Open-ended questions also revealed that most participants found ease of use to be high. Demand and engagement were high as well with a mean number of mobile app entries of 34.1 (SD 20.1) during the postoperative period. There were no reported technological or logistical issues with the mobile app. Participants took an average of 25.13 (SD 14.37) opioid tablets to manage their postoperative pain.

**Conclusions:**

Results of this study revealed that the use of a mobile app for pain and medication management during postoperative recovery was both feasible and acceptable in older veterans undergoing total knee arthroplasty within the Veterans Health Administration. The wide variation in opioid consumption across participants revealed the potential use of the mobile app to provide actionable insights to clinicians if adopted more widely.

## Introduction

### Background

The opioid crisis has resulted in a significant increase in opioid misuse and opioid use disorder; as a result, opioid-related overdose deaths have climbed to staggeringly high levels. Opioid misuse, use disorders, and overdoses are costly in terms of morbidity, mortality, and humanitarian and economic burden. It is estimated that in 2019 alone, more than 9.7 million adults living in the United States (3.5%) misused prescription opioids [1], and drug overdose deaths in the United States rose 29.4% in 2020 to an estimated 93,331, including 69,710 (74.7%) involving opioids [2,3]. Prescription opioid misuse risk is disproportionate among veterans; military veterans wounded in combat misuse prescription opioids at an even higher rate (46.2%) [4].

The cost of opioid overdose, abuse, and dependence in the United States is estimated to be US $78.5 billion annually [5]. If reduced quality of life from opioid use disorder and the value of life lost due to fatal opioid overdose are included, the estimate increases to more than US $1 trillion [6]. Opioid misuse costs the Civilian Health and Medical Program of the Department of Veterans Affairs more than US $1.13 billion annually [6]. Adjusted annual health care costs for diagnosed opioid misuse patients are higher than those for patients without diagnosed misuse, and the prevalence of diagnosed opioid misuse is almost 7 times higher for those in the Veterans Health Administration than in commercial health plans, translating to a significant economic burden for this population [7].

One known risk factor for opioid misuse and dependence is opioid prescription following surgical procedures, both minor and major [8]. Extant data suggest that nonmedical prescription opioid use is a strong risk factor for heroin use initiation among both civilian [9] and veteran populations [10]. For the patient being prescribed opioids, there is a need to ensure appropriate use to prevent habit-forming behaviors during postoperative recovery, as it has been estimated that 12.5% of people who are prescribed opioids misuse them [11] and, as noted earlier, this is even more pronounced among some veterans (46.2%) [4]. For members of the patient’s family and others in the community, it is important that excess prescription opioids are not made available for misuse; indeed, about half (50.8%) of people who misused prescription pain relievers reported obtaining them from a friend or relative, either for free, by purchasing them, or by taking them without asking, and an additional 35.7% got them through a prescription by a single doctor [1]. Several recent studies have also found wide variation in postoperative prescribing practices and noted systemic overprescription [12-14], which may inadvertently promote continued opioid use after their indication is no longer warranted. Taken together, these findings underscore the criticality of managing the use of opioids during the postoperative period in combating the opioid crisis and highlight this time as an essential point of intervention.

### Mobile Health

Over 97% of Americans own cell phones, 85% of which are smartphones. Smartphone ownership is even more common among younger adults; 96% of persons aged between 18 and 29 years, 95% of persons aged between 30 and 49 years, and 83% of persons aged between 50 and 64 years own smartphones [15]. As a result, technology-based health interventions are becoming increasingly common. Among older adults, however, smartphone ownership is less common; among adults over the age of 65 years, smartphone ownership drops to 61% [15].

Mobile health (mHealth) includes everything from the use of mobile technology, such as smartphones, to software, including mobile apps, to facilitate or enhance health care [16]. Mobile technologies are used in multiple ways, including the use of smart apps, web-based software, and SMS text messaging [17]. Smart apps are being used to augment treatment for substance abuse disorders, promote self-management of chronic conditions, increase engagement with health research, assess or measure symptoms, and foster adherence to both treatments and appointments [18-21]. Evidence suggests that symptom reporting with smart apps is well tolerated by patients and has better validity and reliability than hard copies that often rely on recall [22]. One pilot study found that 78% of patients with chronic pain who downloaded an app to track their pain symptom ratings used it with an average of 16.4 daily assessments submitted by patients within the first month. Patients reported that the app was easy to use and were willing to continue to use the app even after the study was complete [22]. Additionally, electronic monitoring (EM) of prescription medication use is a budding technology with a variety of applications and can be integrated into most drug packaging, including pill dispensers and blister packs, to help measure adherence [23]. Prior research has shown that, among veterans receiving outpatient care for posttraumatic stress disorder, age significantly predicted ownership of mHealth devices but not use or interest in mHealth apps among device owners [16]. Therefore, although older veterans may be less likely to own a personal smart device, access should be adequate, and interest in using mHealth apps is high [16].

### CPMRx App

Continuous Precision Medicine has developed a suite of software, CPMRx, that uses scientifically validated tools to help clinicians understand how a patient can use the least amount of medication while getting the most benefit. The suite of software includes a mobile app for patients that includes a behavioral health intervention and a clinical decision support tool for health care providers that provides feedback about pain and medication use trends.

We have used a public health research pipeline approach for designing, evaluating, and implementing behavioral health interventions, and the CPMRx mobile app has been shown to be feasible and acceptable for supporting postoperative pain management across several patient populations and surgery types, including young adults undergoing third molar extraction (dental clinic, Womack Army Medical Center) [24,25], adults undergoing tonsillectomy (ear, nose, and throat, WakeMed Hospital) [26], and women undergoing cesarean section (obstetrics and gynecology, Temple University Health) [27,28]. The effectiveness of the mobile app and clinical decision support tool has also been validated. The implementation of CPMRx provides actionable insights that can be used to establish more precise prescribing guidelines. At Womack Army Medical Center, for example, clinicians were able to reduce the overprescription of an opioid by 10,000 pills annually for a single surgery type (ie, third molar extraction) [24]. In a recently conducted randomized controlled trial at Temple University (N=100), the CPMRx app was shown to reduce the odds of prescription opioid misuse during the postoperative period by 92% [28].

### Specific Aims

The overarching purpose of this single-arm prospective pilot study was to explore whether deploying a mobile app to track postoperative pain and medication use is feasible in a Department of Veterans Affairs Medical Center. In support of this goal, we had four complementary specific aims: (1) determine the technological and logistical feasibility of the mobile app, (2) assess the acceptability of the mobile app to participants, (3) measure demand for and engagement with the mobile app, and (4) explore the potential use of the mobile app to patients and providers.

## Methods

### Participants and Procedures

Participants (N=10) were veterans undergoing total knee arthroplasty at the Charles George VA Medical Center (CGVAMC). Veterans used the CPMRx app to self-manage their pain and track medication use during their 7-day at-home recovery following surgery.

A convenience sample of participants was recruited during presurgical appointments at the CGVAMC. Presurgical appointments are conducted by orthopedic nurse case managers for patients undergoing total knee arthroplasty performed by CGVAMC orthopedic surgeons. During these appointments, patients are educated about the procedure and scheduled for a physical, history, and anesthesia evaluation to ensure they are eligible for the procedure. For this study, in addition to scheduling the physical, history, and anesthesia evaluation, the orthopedic nurse case manager assisted in recruitment by briefing the patient on the research study. The orthopedic nurse coordinator made note of all patients who expressed interest in participating in the research study. During the preoperative appointment, patients provided informed consent and HIPAA (Health Insurance Portability and Accountability Act) authorization.

On the day of discharge from the postsurgical ward, patients typically receive patient education from the registered nurse assigned to them. The discharging provider ensures an order is placed for standard postoperative medications that include opioid pain medication (oxycodone 5 mg every 6 hours as needed or hydromorphone 2 mg every 6 hours as needed, 42 in total) and acetaminophen (650 mg every 6 hours as needed, 84 tablets of 325 mg in total), as well as the other medications typically prescribed following a patient’s respective procedure. For this study, all pain medications provided to study participants were placed into a smart EM blister pack to record time stamps whenever a medication was removed by the patient. All participants received discharge instructions and education as part of the standard of care and additionally received instruction on the mobile app, tablet, and EM blister packs.

Participants self-managed their pain at home and used the mobile app and EM blister packs for the first week (7 days) following discharge to record pain score and medication use, as necessary. Patients met with the orthopedic nurse case manager for a typically scheduled postoperative appointment between 7 and 10 days after surgery. At this appointment, they returned the tablet and EM blister packs and filled out an acceptability survey. All unused medications were disposed of by the study team.

### Software and Hardware

CPMRx software delivers a user-friendly platform that (1) allows users to report dose-by-dose pain scores, (2) helps users consider whether a dose is needed, and (3) creates use traceability. The software allows a user to report their pain score using a modified Visual Analog Scale when a dose is taken and includes a user-directed “gamification” component. This component delivers positive reinforcement cues to the user for managing their pain within recommended treatment protocols with the goal of providing education and incentivizing patients to make smarter and more informed decisions about dose frequency and amount. The software collects and organizes data that can be accessed by clinicians to view trending analysis for pain scores, adherence to treatment plans, and time between doses.

For this study, the mobile app was installed on study-provided smart tablets that were locked into “kiosk mode” (ie, altered to only provide access to the CPMRx mobile app and necessary system functions). The EM pill blister packs used in this study were manufactured by Information Mediary Corporation. Compliance data were extracted from blister packs using a desktop radio frequency identification reader and were then transferred into an electronic record for research purposes.

### Measures

Sociodemographic variables were extracted from patients’ electronic medical records. These included age (in years), sex (male or female), race (White or European American, Black or African American, Asian American, American Indian or Alaska Native, Native Hawaiian or Pacific Islander, 2 or more races, or unknown), current smoking status (smoker or nonsmoker), and history of opioid prescription (dichotomized as yes or no).

Patients self-reported pain within the mobile app on a modified Visual Analog Scale with tick marks at 0-10. The screen reads “How much pain are you in?” and there is a face that changes colors as the user chooses the pain rating on the slider. For those with dexterity issues, the tick marks can simply be tapped.

Acceptability assessments included 2 quantitative measures and 7 open-ended questions. Quantitative measures were ease of use (rated on a 5-point scale from 1=“very hard” to 5=“very easy”) and amount of time required (rated on a 5-point scale from 1=“too much” to 5=“very little”). Open-ended questions sought to better understand the overall user experience and possibilities for improvement (eg, “What did you like most about your experience with the app?” and “How would you improve this app?”). Demand and engagement were measured as the actual use of the mobile app during the postoperative period, and logistical and technological feasibility were assessed by reports of issues with app use. The total number of opioids used was also measured to determine their potential use in informing clinical decision-making.

### Data Analysis

All quantitative analyses were run using SAS (version 9.4; SAS Institute). Univariate statistics were used to summarize both continuous (eg, means, SDs, and medians) and categorical (eg, proportions and total numbers) variables for sample characteristics. Descriptive statistics were run to determine means and SDs of quantitative measures of acceptability.

Qualitative data were coded through thematic analysis using NVivo (version 14; Lumivero). Due to the simplicity of the text, a single researcher coded all open-ended responses and generated themes using an inductive semantic approach.

### Ethical Considerations

This study was approved by the institutional review board at the CGVAMC and was conducted in the performance of a cooperative research and development agreement between Continuous Precision Medicine and the Veterans Health Administration Innovation Ecosystem. All participants provided informed consent and HIPAA authorization. All study data were deidentified. Participants were provided a US $50 gift card for their participation.

## Results

### Sample Characteristics

The sample for this pilot was quite homogeneous; 9 (90%) of the veterans were male, and all 10 (100%) were White nonsmokers with no history of prior opioid prescription. The mean age of the participants was 68.8 (SD 9.7) years with a range of 54 to 81 years.

### Feasibility Outcomes

Overall, quantitative measures of acceptability were high. The average rating for the amount of time required to use the app was 4.88 (SD 0.35; range 4.0-5.0), and the average rating for ease of use was 4.38 (SD 1.06). One participant (age 77 years, male) rated ease of use of the mobile app as hard (2 of 5). Thematic analysis of open-ended questions also revealed ease of use as a central theme, and that most participants found ease of use to be high. For instance, in response to the question, “What did you like most about your experience with the app?” participants cited “ease of documentation,” “very straightforward,” “easy to use,” “easy to understand,” and “increases awareness of medication use and very easy to use.” Demand and engagement were high as well, with a mean number of mobile app entries of 34.1 (SD 20.1) during the postoperative period. There were no reported technological or logistical issues with the mobile app. Participants took an average of 25.13 (SD 14.37) opioid tablets to manage their postoperative pain.

## Discussion

The results of this study revealed that the use of a mobile app for pain and medication management during postoperative recovery was both feasible and acceptable in older veterans undergoing total knee arthroplasty within the Veterans Health Administration. Overall, participants rated ease of use and amount of time required as highly acceptable, with no logistical or technological issues being reported. This is congruent with our prior findings across several studies, including those involving young soldiers undergoing third molar extraction [24,25], adults undergoing tonsillectomy [26], and women undergoing cesarean section [27,28]. This study was the first to include a report by a patient of difficulty using the mobile app, which does warrant discussion. As mentioned previously, a male participant aged 77 years reported that he found the mobile app hard to use and noted in his comments that it was “not intuitive.” Although there is a tutorial embedded within the app, it may be that an additional point of socialization to the software in this population may be helpful. It is our recommendation that the clinical point of contact (eg, the orthopedic nurse case manager) demonstrate how the patient should record information within the app prior to discharge, particularly if the patient reports unfamiliarity with mobile app technologies. This in-person tutorial should mitigate any acceptability concerns for the patient and would, in our estimation, require less than 2 additional minutes of the nurse’s time.

Demand for and engagement with the mobile app were high, which supports prior research among veterans suggesting an interest in adopting mHealth technologies [16]. The number of entries by participants showed that repeated inputs (at each time of medication use) were not too burdensome over the course of the 7-day prescription. In fact, more entries were logged over this 7-day postoperative period than in prior pain studies [22]. Finally, the wide variation in opioid consumption across participants revealed the potential use of the mobile app to provide actionable insights to clinicians if adopted more widely. Taken together, these results suggest that scaling use of the CPMRx mobile app in this population is both feasible and acceptable. Given the small sample size, generalizability of findings is limited. Future research should examine whether these patterns hold across surgical services within the Veterans Health Administration and for longer lengths of care as would be the case for patients dealing with chronic pain. Implementing the CPMRx suite of software may improve clinical care by reducing opioid misuse and preventing habit-forming behaviors at the individual level as well as decreasing excess prescription opioids available for diversion and thereby reducing risk at the community level.

## Abbreviations

CGVAMC: Charles George VA Medical Center
EM: electronic monitoring
HIPAA: Health Insurance Portability and Accountability Act
mHealth: mobile health

## Data Availability

The data sets generated during and/or analyzed during this study are available from the corresponding author on reasonable request.
